# Structural Variation within the Amygdala and Ventromedial Prefrontal Cortex Predicts Memory for Impressions in Older Adults

**DOI:** 10.3389/fpsyg.2012.00319

**Published:** 2012-08-28

**Authors:** Brittany S. Cassidy, Angela H. Gutchess

**Affiliations:** ^1^Department of Psychology, Brandeis UniversityWaltham, MA, USA

**Keywords:** impression formation, aging, memory, amygdala, ventromedial prefrontal cortex

## Abstract

Research has shown that lesions to regions involved in social and emotional cognition disrupt socioemotional processing and memory. We investigated how structural variation of regions involved in socioemotional memory [ventromedial prefrontal cortex (vmPFC), amygdala], as opposed to a region implicated in explicit memory (hippocampus), affected memory for impressions in young and older adults. Anatomical MRI scans for 15 young and 15 older adults were obtained and reconstructed to gather information about cortical thickness and subcortical volume. Young adults had greater amygdala and hippocampus volumes than old, and thicker left vmPFC than old, although right vmPFC thickness did not differ across the age groups. Participants formed behavior-based impressions and responded to interpersonally meaningful, social but interpersonally irrelevant, or non-social prompts, and completed a memory test. Results showed that greater left amygdala volume predicted enhanced overall memory for impressions in older but not younger adults. Increased right vmPFC thickness in older, but not younger, adults correlated with enhanced memory for impressions formed in the interpersonally meaningful context. Hippocampal volume was not predictive of social memory in young or older adults. These findings demonstrate the importance of structural variation in regions linked to socioemotional processing in the retention of impressions with age, and suggest that the amygdala and vmPFC play integral roles when encoding and retrieving social information.

## Introduction

Structural changes to the brain accompany healthy aging (Hedden and Gabrieli, 2004), including cortical thinning, decreased intracranial volume (Salat et al., 2004), and more specific volumetric reductions in subcortical structures (Walhovd et al., 2005). These structural changes manifest in behavioral differences when compared to healthy younger adults. For instance, reductions in hippocampal volumes correspond with age-associated visual and verbal memory impairments (Soininen et al., 1994), age-related decrements in explicit memory performance (Raz et al., 1998), and longitudinal changes on aging-sensitive memory tests (Golomb et al., 1996).

Although aging also affects functional engagement of the hippocampus, including working (Mitchell et al., 2000), and episodic (Daselaar et al., 2003) memory processes, recent research suggests the existence of a functional neural mechanism underlying memory for social information, and that involves the recruitment of medial prefrontal cortex in contrast to the hippocampus (Mitchell et al., 2004; Gilron and Gutchess, 2012). Regions within this “social” memory system (e.g., dorsal and ventral medial prefrontal cortex) are recruited when learning and remembering social material, such as autobiographical memories (Gilboa, 2004), self- versus other-related items (Kelley et al., 2002), and impressions (Mitchell et al., 2004; Gilron and Gutchess, 2012). Like the relationship between hippocampal atrophy and memory for non-social material in healthy aging, social memory may be sensitive to age-related structural changes to more “social” brain regions. Thus, the relative integrity of these regions may be associated with the level of remembered social information in older, but not necessarily younger, adults.

Recent neuroimaging studies have begun to investigate how aging affects the neural underpinnings of social processing. These studies have found age-invariant neural recruitment in several social tasks, including self-referencing (Gutchess et al., 2007), theory of mind (Castelli et al., 2010), reaction to social affiliation and isolation (Beadle et al., 2012), and social evaluation (Cassidy et al., in press). Some age differences in neural recruitment in response to social stimuli have been identified, such as in the elaborative encoding of self-related information (Gutchess et al., 2010), and in mentalizing tasks (Moran et al., 2012). Thus, functional engagement of “social” brain regions may be intact in healthy aging to an extent. For instance, in easy tasks (Castelli et al., 2010) or tasks requiring a consideration of the self (Gutchess et al., 2007; Cassidy et al., in press), function within neural regions underlying social processing may be relatively spared. However, more difficult tasks or tasks that do not garner self-involvement may not similarly engage these regions in older adults (Moran et al., 2012).

Behavioral work evidences relative age-related sparing of social memory processes. In contrast to studies reporting age-related decline on hippocampally dependent memory tasks (Squire, 1992; Grady et al., 2003; Dennis et al., 2008), other work evidences that older and younger adults similarly remember socioemotional information (Rahhal et al., 2002; May et al., 2005; Cassidy and Gutchess, 2012). In the present study, we focused on impression formation, an interpersonally relevant domain where older adults may be motivated to utilize and remember information. For example, older adults may be more sensitive than young adults to cues that can modify an initial first impression, as well as the diagnosticity of traits (Hess and Auman, 2001; Hess et al., 2005). Younger and older adults may similarly remember impressions (Todorov and Olson, 2008), predominantly when impressions are formed in a context emphasizing interpersonal relationships (Cassidy and Gutchess, 2012). Choosing a domain where older adults may not exhibit poorer memory than younger adults allows us to better examine if structural variation differentially affects memory in older over younger adults.

Considering the impact of structural variation within the neural regions underlying social processing offers a complementary approach to neuroimaging and behavioral studies to examine the preservation of memory for impressions with age. Such an approach could examine if older adults with more structural atrophy and thinning exhibit *poorer* social memory performance relative to older adults with less structural change. Previous research using healthy young adults and lesioned individuals has linked structural integrity of two regions implicated in impression formation and social evaluation, the amygdala (Todorov and Olson, 2008), and ventromedial prefrontal cortex (vmPFC; Milad et al., 2005), to socioemotional memory. Interestingly, vmPFC receives substantial input from the amygdala, and these regions are linked in several socioemotional processes, including emotional memory (Phelps et al., 2004) and reward expectancy and choice (Hampton et al., 2007). This relationship suggests that age-related structural variation within these regions may also extend to memory for impressions.

The amygdala is widely implicated in impression formation (Schiller et al., 2009; Baron et al., 2010) and its integrity is critical when remembering impressions (Todorov and Olson, 2008). Intact amygdala volume is also necessary for retrieving socially relevant information in response to visual stimuli (Adolphs et al., 1998), suggesting that among non-lesioned individuals, structural variation within the amygdala might correspond with the ability to remember impressions when viewing individuals previously paired with trait-inferring behaviors. Although results are mixed as to whether the amygdala undergoes significant age-related atrophy (Soininen et al., 1994; Jack et al., 1997; Good et al., 2001; Allen et al., 2005), potentially smaller amygdala volumes in older compared to younger adults might lead to differential behavioral performance. The relationship between amygdala volume and memory for impressions may be more apparent in older over younger adults, given that the sensitivity to detect behavioral changes may not manifest without substantial structural atrophy (Raz et al., 1998).

In younger adults, thicker vmPFC corresponds with enhanced extinction retention after a fear conditioning task (Milad et al., 2005). In addition, the relationship between vmPFC thickness and emotional learning correlates with extraversion, a personality characteristic that influences the social situations in which an individual will or will not participate (Rauch et al., 2005). vmPFC activity is implicated in numerous social processes, including the learning of social information (Behrens et al., 2008), empathy (Shamay-Tsoory, 2011), the analysis of social content (Schilbach et al., 2006), and social evaluation (Cassidy et al., in press). Although research documents overall age-related cortical thinning (Fjell et al., 2009), vmPFC may not undergo such stark structural changes (Salat et al., 2001). Even though older adults may experience relatively less vmPFC thinning compared to other regions, structural variation within this region might differentially affect performance on tasks engaging that area regardless of age, based on evidence that thickness in young adults relates to emotional memory performance (Milad et al., 2005), and this finding could extend to social memory tasks. More specifically, the integrity of this region may be critical in remembering valuable social information (e.g., aversive stimuli within a fear extinction paradigm) regardless of age. However, although memory for valuable social information may relate to vmPFC thickness in both younger and older adults, this relationship may be particularly prominent in an older population, who place more emphasis on personally salient socioemotional material than young (Fredrickson and Carstensen, 1990; Carstensen and Turk-Charles, 1994; Carstensen et al., 1999).

The current study investigated how structural variation within regions important to socioemotional (amygdala, vmPFC) and explicit (hippocampus) memory affects the retrieval of impressions in healthy aging. We predicted that older adults would have smaller amygdala and hippocampal volumes relative to young, but that there would not be an overall age difference in vmPFC thickness. We expected amygdala volume to be predictive of memory for impressions regardless of the context in which the impressions were formed, given the amygdala’s widespread role in impression formation. We anticipated older adults would drive this relationship, given the expectation of the association with memory would be more pronounced for smaller amygdala volumes. We did not expect hippocampal volume to be predictive of memory for impressions, given lesion work showing that the hippocampus is not necessary to learn and retain person information (Todorov and Olson, 2008). If hippocampal lesion patients can successfully encode and retrieve impressions, age-related atrophy should also be unrelated to this ability. Finding a relationship between amygdala, and not hippocampal, integrity, and social memory would provide evidence for the existence of a social memory system potentially separable from hippocampally dependent memory systems. Given that vmPFC is engaged when processing socially meaningful information (e.g., self-related material), we expected that vmPFC thickness would predict memory for impressions in younger and older adults, but primarily in a context having significant social value. Because older adults are particularly sensitive to emotionally meaningful information, we anticipated that structural variation among older adults would drive this relationship.

## Materials and Methods

### Participants

Fifteen older (61–85 years old, six males; *M* = 72.80, SD = 6.91) and 15 younger (20–29 years old, eight males; *M* = 21.13, SD = 3.00) adults recruited from Brandeis University and the surrounding community participated. The Brandeis University and Partners Healthcare institutional review boards approved this study, and participants provided written informed consent. Older adults were screened for cognitive orientation with MMSE scores >26 (Folstein et al., 1975; *M* = 29.07, SD = 1.33) to ensure no significant cognitive impairment, and were characterized on cognitive measures to assess comparability to others in the literature. Age groups had similar years of education and vocabulary scores (Shipley, 1986). Young adults had faster processing speed (*M* = 83.60, SD = 14.07) than older adults (*M* = 53.87, SD = 9.06), *t*(28) = 6.88, *p* < 0.001, using a digit-comparison measure (Hedden et al., 2002), and had higher letter-number sequencing scores (Wechsler, 1997; *M* = 12.60, SD = 2.80) than older adults (*M* = 10.40, SD = 2.77), *t*(28) = 2.16, *p* = 0.04.

### Stimuli

Ninety-six images of Caucasian faces (evenly distributed across young/old and male/female) with neutral expressions, and rated for attractiveness, distinctiveness, and trustworthiness (Gilron and Gutchess, 2012), were drawn from the PAL database (Minear and Park, 2004). Each face was paired with a unique trait-inferring behavioral sentence, drawn from a dataset (Uleman, unpublished data) previously rated for trait convergence, arousal, and valence extremity by young and older adults (Cassidy and Gutchess, 2012). Forty-eight sentences inferred positive traits and 48 inferred negative traits.

### Procedure

Participants were told they would be forming impressions and making judgments of others. Participants practiced the task, receiving feedback on their responses, before completing the full task in the scanner. Description of functional data obtained from this task are reported elsewhere (Cassidy et al., in press). Stimuli were presented via E-Prime software (Psychology Software Tools, Pittsburgh, PA, USA).

**Figure 1:**
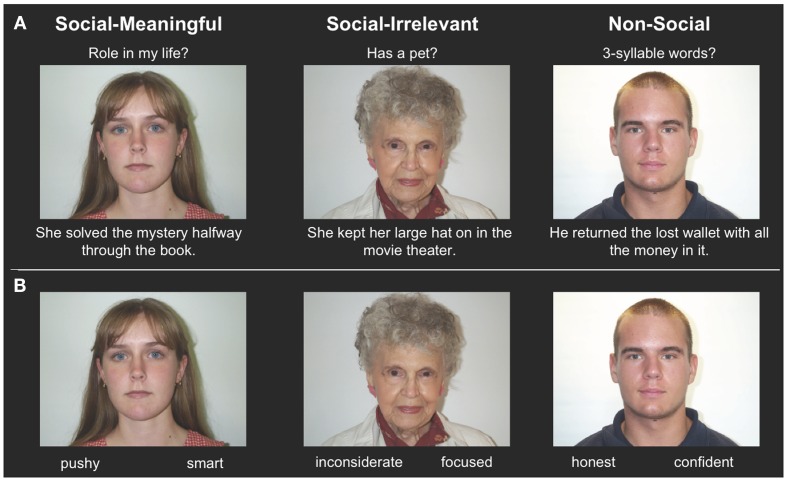
**(A)** Example encoding stimuli, showing the three evaluation types (social-meaningful, social-irrelevant, and non-social) with example face-behavior pairs. The evaluation types were not explicitly labeled on the screen, and participants answered yes or no to the displayed prompt. **(B)** Example retrieval stimuli, showing examples of target and lure traits.

Participants encoded 96 trait-inferring face-behavior pairs one at a time for 6000 ms. Participants were instructed to form impressions based on the face-behavior pairs, and then to answer the prompt displayed on top of the display (Figure 1A). One-third of the trials directed participants to the social-meaningful evaluation (“Do I want this person to play a role in my life?”), one-third to the social-irrelevant evaluation (“Does this person have a pet?”), and one-third to the non-social evaluation (“Does the sentence contain any three syllable words?”). Participants responded “yes” or “no” to the prompts via button box. Sentences of positive and negative valence, along with the four age-gender groups, were evenly distributed among the three evaluations. Attractiveness, distinctiveness, and trustworthiness ratings of faces did not differ by evaluation condition. Trials were interspersed with periods of delay ranging from 2000 to 20,000 ms (indicated by a fixation point at the center of the screen). These intervals were obtained using the Optseq program^1^.

There was an approximately 7 min retention interval where participants did not perform any task. Participants then completed a self-paced retrieval task outside of the scanner (Figure 1B). All previously viewed faces were presented in one block, one at a time in a random order. Two trait adjectives were listed below each face. One was the correct response, inferred from the encoding behavior, and the other was a non-inferred lure unrelated to the target trait. Target traits were the most commonly generated impressions from norms (Uleman, unpublished data), and lure traits were experimenter-generated. Participants indicated which trait they remembered as associated with the face. Half of the presented lure traits had matching valence of the inferred trait without being synonyms (e.g., friendly versus generous), and half had unmatched valence of the inferred trait, but were not antonyms (e.g., friendly versus dull). Participants then completed additional cognitive measures.

### Anatomical data acquisition

Data was collected via a Siemens Trio 1.5 T whole-body scanner (Siemens Medical Systems, Iselin, NJ, USA). High-resolution T1-weighted anatomical images were acquired using a multiplanar rapidly acquired gradient echo (MP-RAGE) sequence. All anatomies were reconstructed using FreeSurfer 5.0^2^ running on CentOS 5.

#### Measurement of subcortical volume in individual participants

**Figure 2:**
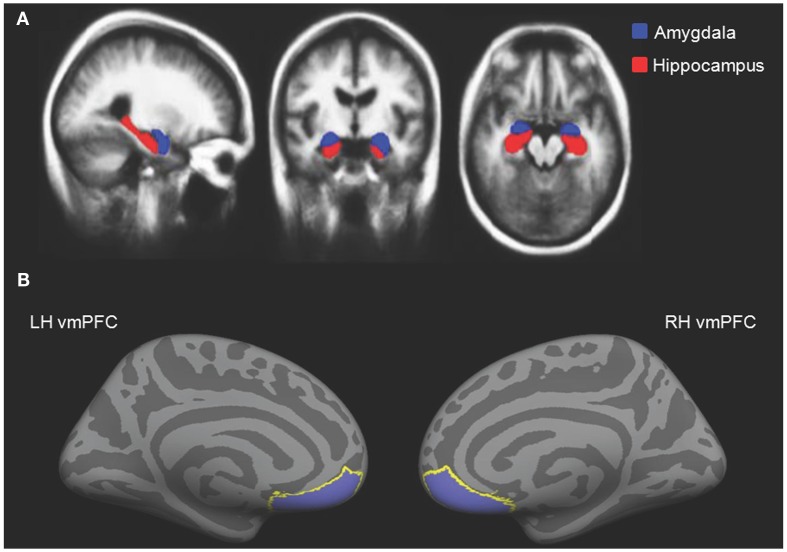
**(A)** Independently defined anatomical volumetric ROIs of bilateral amygdala and hippocampus and **(B)** independently defined anatomical surface-based ROIs of bilateral vmPFC.

To assess amygdala and hippocampal volumes, we performed a quantitative analysis of T1-weighted MRI data using an automated segmentation technique widely used in volumetric studies (McDonald et al., 2008; Bickart et al., 2010). This method uses a manually labeled atlas dataset from 40 individuals to automatically segment and assign anatomical region-of-interest (ROI) labels to 40 different brain structures, including our *a priori* ROIs of the amygdala and hippocampus (Figure 2A). Regions are labeled based on probabilistic estimations, and the method is comparable to manual labeling (Fischl et al., 2002). Because subcortical volumes vary with head size, we performed our statistical analyses using amygdala and hippocampal volumes corrected for individual intracranial volume, a technique used in previous volumetric studies (O’Brien et al., 2006; Wright et al., 2006), including research in aging individuals (Walhovd et al., 2010; Jackson et al., 2011).

#### Measurement of cortical thickness in individual participants

To assess cortical thickness, we used the FreeSurfer surface-based analysis software tools, a method previously described in detail (Dale and Sereno, 1993; Fischl et al., 1999; Fischl and Dale, 2000). To summarize the technique, the anatomical scan for each participant was first used to segment cerebral white matter and to estimate the gray-white interface. Topological defects in this estimate were inspected by an experimenter and manually corrected, as needed, and this estimate was used as a starting point for a surface algorithm designed to obtain precise measurement of the pial surface. The cortical surface in each participant was then visually inspected for inaccuracies in segmentation. Next, thickness measures across the cortex were computed by finding the point on the gray-white interface that was closest to a given point on the estimated pial surface and averaging between these values in each participant (Fischl and Dale, 2000). The accuracy of this technique to obtain cortical thickness has been previously validated by comparisons with manual analysis on postmortem brains (Rosas et al., 2002), as well as direct comparisons with anatomical MRI data (Kuperberg et al., 2003), and has been used in research conducted in aging individuals (Desikan et al., 2009; Fjell et al., 2009). Our *a priori* ROIs for vmPFC were defined using the automatically delineated labels for left and right “medial orbitofrontal cortex” within FreeSurfer (Figure 2B; Fischl et al., 2004; Desikan et al., 2006).

## Results

### Behavioral data

We analyzed participants’ accuracy (proportion of correct responses in remembering impressions) in the retrieval task using a 2 × 3 ANOVA with Age Group (young, old) as a between-groups factor and Evaluation (non-social, social-irrelevant, social-meaningful) as a within-group factor. See Table 1 for a breakdown of performance by age group and evaluation type. There was a main effect of Age Group, *F*(1, 28) = 5.58, *p* = 0.03, $\eta_{p}^{2}=0.17$. Young adults had increased retrieval accuracy (*M* = 63.82%, SD = 7.23%) over older adults (*M* = 57.22%, SD = 7.90%). There was also a main effect of Evaluation, *F*(2, 56) = 8.38, *p* = 0.001, $\eta_{p}^{2}=0.23$. Contrasts showed that participants had better memory for impressions formed when making the social-meaningful evaluations (*M* = 64.27%, SD = 11.53%) than the non-social evaluations (*M* = 54.90%, SD = 9.40%), *F*(1, 28) = 14.67, *p* = 0.001, $\eta_{p}^{2}=0.34$. Participants also had better memory for impressions formed when making the social-irrelevant evaluations (*M* = 62.30%, SD = 12.50%) over the non-social evaluations, *F*(1, 28) = 8.45, *p* = 0.01, There was no difference in memory performance for impressions formed when making the social-meaningful versus social-irrelevant evaluations, *F*(1, 28) < 1, ns. There was no Age Group by Evaluation interaction, *F*(2, 56) = 1.40, ns.

**Table 1:** **Retrieval test accuracy (M, SD) for each age group split by evaluation type**.

	Younger adults (*N* = 15)	Older adults (*N* = 15)	*t*-Statistic	*p*-Value
Social-meaningful	65.21% (10.62%)	63.33% (12.69%)	0.44	0.66
Social-irrelevant	66.67% (10.48%)	57.92% (13.15%)	2.02	0.05
Non-social	59.38% (9.30%)	50.42% (7.07%)	2.97	0.01

### Behavioral correlations with amygdala and hippocampal volume and vmPFC thickness

To assess overall positive relationships between structural variation and social memory, we examined the relationships between left and right hippocampal and amygdala volume (corrected for intracranial volume), as well as left and right vmPFC thickness, with overall retrieval accuracy in the memory test, while controlling for age. We also examined the relationship between structural variations in these regions with retrieval accuracy for impressions formed in each evaluation condition, while controlling for age. To assess whether young or older adults predominantly drove these relationships, we again calculated these relationships separately for each age group. We assessed the significance of the difference between Pearson correlation coefficients for young and older adults using the Fisher *r*-to-*z* transformation. Because we predicted positive correlations between volume and thickness with memory, one-tailed Fisher *r*-to-*z* transformations were used. All correlations reported for the amygdala and hippocampus were calculated after correcting for intracranial volume.

#### Amygdala

**Figure 3:**
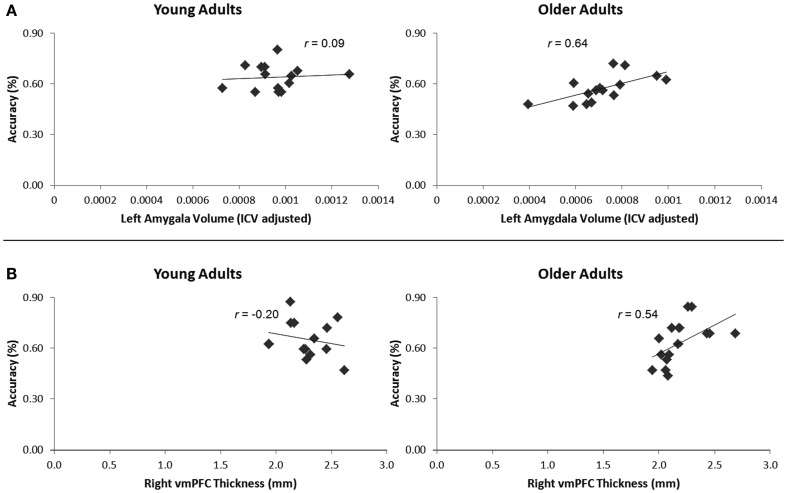
**(A)** Increasing left amygdala volume, corrected for intracranial volume, corresponded with enhanced overall memory for impressions in older, but not younger adults. **(B)** Increasing right vmPFC thickness corresponded with enhanced memory for impressions formed when making the social-meaningful evaluations in older, but not younger adults.

Older adults had smaller left amygdala volumes (range: 596–1526 mm^3^, *M* = 1107.67 mm^3^, SE = 62.46) than young (range: 1167–1904 mm^3^, *M* = 1481.67 mm^3^, SE = 44.63), *t*(28) = 4.87, *p* < 0.001. Older adults also had smaller right amygdala volumes (range: 826–1655 mm^3^, *M* = 1238.13 mm^3^, SE = 60.17) than young (range: 1227–1841 mm^3^, *M* = 1532.07 mm^3^, SE = 55.63), *t*(28) = 3.59, *p* = 0.001. These differences held when correcting for intracranial volume, *p*s < 0.001. When corrected for intracranial volume, there was a significant positive relationship between left amygdala volume and overall memory for impressions, controlling for age, *r*(27) = 0.43, *p* = 0.02, 95% CI [0.18, 0.64]. To assess whether structural variation within older adults primarily drove this relationship, we calculated this correlation split by age group. Left amygdala volume positively correlated with overall memory for impressions in older, *r*(13) = 0.64, *p* = 0.01, 95% CI [0.41, 0.85] but not younger adults, *r*(13) = 0.09, ns (Figure 3A). The difference between these two correlations was marginally significant, *z* = 1.64, *p* = 0.05. There was also a significant positive relationship between left amygdala volume and memory for impressions formed when making non-social evaluations, controlling for age, *r*(27) = 0.43, *p* = 0.02, 95% CI [0.07, 0.70]. However, when split by age group, this correlation was not significant among older or younger adults, *p*s > 0.10. Additionally, there was a positive relationship between left amygdala volume and memory for impressions formed when making the social-irrelevant evaluations in older adults, *r*(13) = 0.51, *p* = 0.05, 95% CI [−0.01, 0.85] whereas this relationship was not apparent among young adults, *r*(13) = −0.23, ns. The difference between these two correlations was significant, *z* = 1.95, *p* = 0.03. Controlling for age, there were no positive correlations found between right amygdala volume and memory for impressions, neither overall or in any of the three evaluation conditions. When split by age group, no positive relationships emerged.

#### Hippocampus

Older adults had smaller left hippocampus volumes (range: 2382–4072 mm^3^, *M* = 3109.40 mm^3^, SE = 112.11) relative to young (range: 3224–4656 mm^3^, *M* = 3921.73 mm^3^, SE = 106.53), *t*(28) = 5.25, *p* < 0.001. Older adults also had smaller right hippocampus volumes (range: 2532–4011 mm^3^, *M* = 3200.60 mm^3^, SE = 111.22) than young (range: 3250–4365 mm^3^, *M* = 3859.87 mm^3^, SE = 94.93), *t*(28) = 4.51, *p* < 0.001. These differences held when correcting for intracranial volume, *p*s < 0.001. When corrected for intracranial volume and controlling for age, there were no significant correlations between left or right hippocampal volume and overall memory for impressions or memory performance in any of the three evaluation conditions. When split by age group, there were also no significant correlations between increasing left or right hippocampal volume and overall memory or memory in any of the three evaluation conditions in young or older adults.

#### vmPFC

Older adults had thinner left vmPFC (range: 2.12–2.49 mm, *M* = 2.27 mm, SE = 0.03) relative to young (range: 2.16–2.79 mm, *M* = 2.41 mm, SE = 0.05), *t*(28) = 52.38, *p* = 0.03. However, older adults did not have thinner right vmPFC (range: 1.94–2.69 mm, *M* = 2.19 mm, SE = 0.05) than young (range: 1.93–2.62 mm, *M* = 2.29 mm, SE = 0.05), *t*(28) = 1.33, *p* = 0.19.

When controlling for age, neither right nor left vmPFC thickness corresponded with memory for impressions formed in the interpersonally meaningful condition. However, given our *a priori* hypothesis that impressions formed in a socially meaningful context might be particularly salient for older over younger adults, we calculated correlations split by age group. In older adults, right vmPFC thickness positively correlated with memory for impressions formed when making social-meaningful evaluations, *r*(13) = 0.54, *p* = 0.04, 95% CI [0.30, 0.82], whereas this relationship was not evident among younger adults, *r*(13) = −0.20, ns (Figure 3B). The difference between these correlations was significant, *z* = −1.96, *p* = 0.02. When controlling for age, there were no positive correlations between left or right vmPFC thickness and overall memory for impressions or memory for impressions formed when making the social-irrelevant or non-social evaluations. When split by age group, there were also no positive correlations between increasing left or right vmPFC thickness and overall memory or memory for impressions formed when making the social-irrelevant or non-social evaluations.

## Discussion

This study investigated the possibility that older adults’ ability to remember impressions might be associated with structural variation within brain regions previously implicated in socioemotional memory (vmPFC and amygdala) but not a region implicated in explicit memory (hippocampus), whereas young adults’ memory performance might be less affected by structural variability. Although previous research has implicated hippocampal atrophy as being associated with behavioral performance on memory tasks (Golomb et al., 1993, 1996; Soininen et al., 1994; Raz et al., 1998), other work suggests the existence of a social memory system potentially separable from the hippocampally dependent explicit memory system (Mitchell et al., 2004). Although the neural underpinnings of impression formation may be relatively spared with age (Cassidy et al., in press), the structural integrity of regions implicated in these processes may affect the extent of successful retrieval of impressions. We show that variation in amygdala volume and vmPFC thickness corresponds with the extent of successfully retrieved impressions in older, but not younger adults, such that less structural atrophy in amygdala volume and thicker vmPFC are related to enhanced memory for impressions. In contrast, relative hippocampal volume did not correspond with memory for impressions, suggesting that social memory may be less affected by the structural integrity of the hippocampus, although additional research must replicate and expand upon this null finding. Prior research has demonstrated that lesions to the amygdala (Adolphs et al., 1998, 2005; Anderson and Phelps, 2001; Todorov and Olson, 2008) and vmPFC (Shamay-Tsoory et al., 2003, 2007; Koenigs and Tranel, 2007; Young et al., 2010) have many behavioral consequences for social cognition. Importantly, this study extends this literature to the structural changes accompanying healthy aging.

In older adults, left amygdala volume positively correlated with overall memory for impressions, whereas this relationship was not observed in the younger cohort. Previous research has shown that individuals with medial temporal lobe lesions extending into the amygdala and temporal pole have difficulty retrieving impressions of others (Todorov and Olson, 2008). Although the study also reported equivalent memory for impressions of others across younger and older adults, there was, however, a notably wide range in performance among the age groups, allowing for the possibility that structural variation in regions such as the amygdala could be associated with the level of memory performance. The current work suggests that variation in performance in older adults may depend in part on the extent of left amygdala atrophy. While some older adults may indeed remember impressions to the same extent as young in some circumstances (Cassidy and Gutchess, 2012), the integrity of the amygdala may correspond with the extent of age-related preservation. It is also noteworthy that, among all participants, left amygdala volume still positively correlated with retrieval of impressions when controlling for age. This suggests that regardless of age, left amygdala volume is critical in determining how well individuals remember impressions.

Moreover, it may be that amygdala volume may begin to affect memory for impressions once atrophy has passed a particular threshold, similar to the idea that the link between structural integrity and behavioral performance may not become apparent until brain regions lose a substantial portion of their volumes. For instance, Raz et al. (1998) found no relationship between limbic region structure and explicit memory until limiting their analysis to a subsample of individuals over 60 years old, where more age-related structural atrophy would be expected compared to a younger cohort. Differences in the extent of medial temporal lobe atrophy have also been shown to dissociate memory performance among individuals with probable Alzheimer’s disease from age-matched controls (Scheltens et al., 1992). This may explain why in the current study, the relationship between amygdala volume and memory for impressions persisted among older, but not younger, adults.

Notably, this relationship was observed in the left, but not the right, amygdala. Left amygdala engagement has been implicated in the encoding of verbal affective information and detailed feature extraction, whereas right amygdala activity is involved in the retrieval of emotional visual information (Markowitsch, 1998). Because our retrieval task required participants to reflect on previously learned socioemotional verbal information, given the role of the left amygdala in encoding affective verbal information, it could indicate that left amygdala integrity would be particularly sensitive to the retrieval of impressions formed off the basis of verbal material. However, some work has evidenced that both left and right amygdala volumes are correlated with visual, but not verbal memory in aging (Soininen et al., 1994). The contribution of the amygdala bilaterally may be more pronounced for non-social tasks, whereas the present task, with its heavy emphasis on verbal information about behavior, may be more sensitive to the relative integrity of the left amygdala.

We also found that increasing right vmPFC thickness corresponded with enhanced memory for impressions formed in the socially meaningful context in older, but not younger adults. This was contrary to our hypothesis that both age groups’ memory for impressions would be related to vmPFC thickness, given that previous fear extinction work in young adults showed that vmPFC thickness predicts emotional memory retention (Milad et al., 2005). Fear extinction work relies on a physiological reaction as evidence of prior learning, and not an explicit memory task as in the current study; thus the nature of retrieved information differs between the tasks. While some face stimuli had negative impressions associated with them, being asked to retrieve information about these individuals would not bring back memory of a painful experience, as in fear extinction work. Given that older adults have an increased focus on socioemotionally meaningful material relative to young adults, who have an overall information acquisition focus (Carstensen and Turk-Charles, 1994; Carstensen et al., 1999; Carstensen and Mikels, 2005), it might seem unsurprising that the relationship between vmPFC integrity and memory for impressions formed in a socially meaningful context was stronger for older compared to younger adults.

Interestingly, the relationship between right vmPFC integrity and impression memory occurred despite the fact that there was no age-related difference in right vmPFC thicknesses overall, and when combining the cohorts and controlling for age, the relationship between thickness and memory did not hold. While amygdala volume moderated overall memory for impressions in older adults, the extent of vmPFC thickness may play a more nuanced role in the ability to remember information older adults consider particularly valuable (e.g., impressions formed in a socially meaningful context). Lesion research has suggested that processes associated with vmPFC optimize decision-making processes by encoding a future goal’s abstract value (Moretti et al., 2009). In the current work, older adults’ right vmPFC thickness corresponded with memory for more impressions that had been formed when making a socially meaningful evaluation, consistent with the idea that older adults prioritize incoming socioemotional material (Carstensen et al., 1999). The vmPFC’s role in memory was not evident among younger adults, perhaps because their overall focus on acquiring knowledge means that they value novel information regardless of the particular evaluation they make.

One limitation of the current work is the relatively small sample size for young and older adults (*N*s = 15). Smaller sample sizes may not be substantial enough to reflect the large variations in brain structure seen in older adult cohorts (Raz and Rodrigue, 2006), particularly when capturing age differences in a cross-sectional, rather than longitudinal, design (Raz et al., 2005). Thus, while the present data may be considered preliminary evidence that structural variation within the amygdala and vmPFC, but not the hippocampus, leads to age differences in remembering impressions, null effects may be a result of small sample size, or a limitation of cross-sectional design. A more sensitive way for future research to estimate these differences would be to assess whether intraindividual structural change relates to age differences in memory, as previous research has shown that within-individual structural change is sensitive to cognition to a greater extent than cross-sectional estimates (Rodrigue and Raz, 2004).

Recently, cognitive neuroscience researchers have illustrated that low statistical power (Yarkoni, 2009) can lead to misleading correlations between brain activity and human behavior. It is important to note that the regions of interest in the current work were anatomically defined, and that anatomical information was correlated with human behavior using a similar methodology as prior work sensitive to these concerns (Bickart et al., 2010). Correlating our behavioral data with anatomically defined regions of interest rather than functionally defined regions from previous analyses allows for the memory and neural measures to be considered independent. Further, despite our limited sample size, our hypotheses were *a priori* and derived from previous work demonstrating how amygdala and vmPFC integrity affect different aspects of social cognition. Nevertheless, it is important to consider the current study as preliminary evidence that amygdala and vmPFC integrity influence memory for impressions in older adults, and further work is needed.

Although this work may serve as a basis for future research, it is important for future studies to consider using samples with a full range of ages across the lifespan, instead of two distinct age groups. This may allow for greater variability in both volumetric and thickness measurements, as well as greater variation in memory performance. Future work might also consider different types of social memory. While the current work tested explicit memory for impressions, the integrity of social cognition regions may be particularly important in a more difficult memory task (e.g., free recall). It would also be of use for future studies to contrast social against non-social memory tasks, which would be expected to rely on the hippocampus. Showing dissociation between how the integrity of regions involved in social versus non-social cognition affect social and non-social memories, respectively, may further substantiate claims that social and non-social explicit memory rely on distinct neural substrates.

In summary, these findings are initial evidence that structural variation in amygdala volume and vmPFC thickness influence the extent to which older adults are able to successfully retrieve impressions. Moreover, the data provide preliminary support for the existence of a social memory system potentially separable from hippocampally dependent systems, as hippocampal integrity was not shown to predict memory for impressions in young or older adults despite significant structural atrophy in older adults compared to young. Although some research has shown that regions implicated in impression formation and social evaluation are functionally relatively spared with age (Cassidy et al., in press), the current study complements this work by showing that the integrity of regions involved in social processing and memory matter as well. Future work can clarify this relationship by testing how structural variation influences the accuracy of different types of social decisions (e.g., appropriate approach behavior in the face of a previously seen unsafe individual). Such work is critical, as our memories of others profoundly impact our social judgments and behaviors throughout the lifespan.

## Conflict of Interest Statement

The authors declare that the research was conducted in the absence of any commercial or financial relationships that could be construed as a potential conflict of interest.

## Footnotes

^1^http://surfer.nmr.mgh.harvard.edu/opt-seq

^2^http://surfer.nmr.mgh.harvard.edu
